
JOURNAL INFORMATION
==============================
NLM Title Abbreviation: Natl Sci Rev
Journal ID: Natl Sci Rev
NLM Journal ID: 101633095
Journal ID: nsr

National Science Review

ISSN: 2095-5138
EISSN: 2053-714X

ARTICLE INFORMATION
==============================
PMCID: PMC7665622
PMID: 34676091
DOI: 10.1093/nsr/nwaa257
Article ID: nwaa257
Article version: 1
Subjects: Letters, AcademicSubjects/MED00010, AcademicSubjects/SCI00010

A proposal for clinical trials of COVID-19 treatment using homo-harringtonine

Wen Hai-Jun State Key Laboratory of Biocontrol, School of Life Sciences, Sun Yat-sen University, Guangzhou 510275, China

Liu Feng-Liang Key Laboratory of Animal Models and Human Disease Mechanisms of Chinese Academy of Sciences / Key Laboratory of Bioactive Peptides of Yunnan Province, Kunming Institute of Zoology, Chinese Academy of Sciences, Kunming, Yunnan 650223, China
Kunming National High-level Bio-safety Research Center for Non-human Primates, Center for Biosafety Mega-Science, Kunming Institute of Zoology, Chinese Academy of Sciences, Kunming, 650107, China

Huang Ming-Xing Department of Infectious Diseases, The Fifth Affiliated Hospital, Sun Yat-sen University, Zhuhai, Guangdong, China

Luo Rong-Hua Key Laboratory of Animal Models and Human Disease Mechanisms of Chinese Academy of Sciences / Key Laboratory of Bioactive Peptides of Yunnan Province, Kunming Institute of Zoology, Chinese Academy of Sciences, Kunming, Yunnan 650223, China
Kunming National High-level Bio-safety Research Center for Non-human Primates, Center for Biosafety Mega-Science, Kunming Institute of Zoology, Chinese Academy of Sciences, Kunming, 650107, China

He Wen-Bin State Key Laboratory of Genetic Resources and Evolution, Kunming Institute of Zoology, Chinese Academy of Science, Kunming 650223, China
Center for Excellence in Animal Evolution and Genetics, Chinese Academy of Sciences, Kunming 650223, China

Feng Jing State Key Laboratory of Genetic Resources and Evolution, Kunming Institute of Zoology, Chinese Academy of Science, Kunming 650223, China
Center for Excellence in Animal Evolution and Genetics, Chinese Academy of Sciences, Kunming 650223, China

Chen Fang-Liang Kunming Police Dog Base of the Ministry of Public Security, Kunming, China

Cai Qi-Chun Cancer Center, Clifford Hospital, Jinan University, Guangzhou, PR China

Ma Hua-Juan Cancer Center, Clifford Hospital, Jinan University, Guangzhou, PR China

Yang Zi-Feng National Clinical Research Center for Respiratory Disease, Guangzhou Institute of Respiratory Health, First Affiliated Hospital of Guangzhou Medical University, State Key Laboratory of Respiratory Disease (Guangzhou Medical University), Guangzhou, China
Faculty of Chinese Medicine, Macau University of Science and Technology, Taipa, Macau SAR, PR China

Zhou Xi State Key Laboratory of Virology, Wuhan Institute of Virology, Center for Biosafety Mega-Science, Chinese Academy of Sciences (CAS), China
Center for Translational Medicine, Wuhan Jinyintan Hospital, China
Joint Laboratory of Infectious Diseases and Health, Wuhan Institute of Virology & Wuhan Jinyintan Hospital, CAS, China

http://orcid.org/0000-0003-4097-6131 Shang You Joint Laboratory of Infectious Diseases and Health, Wuhan Institute of Virology & Wuhan Jinyintan Hospital, CAS, China
Department of Critical Care Medicine, Union Hospital, Tongji Medical College, Huazhong University of Science and Technology, Wuhan, China

Lyu Xue-Mei State Key Laboratory of Genetic Resources and Evolution, Kunming Institute of Zoology, Chinese Academy of Science, Kunming 650223, China
Center for Excellence in Animal Evolution and Genetics, Chinese Academy of Sciences, Kunming 650223, China

Zhang Ding-Yu Center for Translational Medicine, Wuhan Jinyintan Hospital, China
Joint Laboratory of Infectious Diseases and Health, Wuhan Institute of Virology & Wuhan Jinyintan Hospital, CAS, China

Xiao Fei Department of Infectious Diseases, The Fifth Affiliated Hospital, Sun Yat-sen University, Zhuhai, Guangdong, China
Guangdong Provincial Key Laboratory of Biomedical Imaging and Guangdong Provincial Engineering Research Center of Molecular Imaging, The Fifth Affiliated Hospital, Sun Yat-sen University, Zhuhai, Guangdong Province, China

Shan Hong Guangdong Provincial Key Laboratory of Biomedical Imaging and Guangdong Provincial Engineering Research Center of Molecular Imaging, The Fifth Affiliated Hospital, Sun Yat-sen University, Zhuhai, Guangdong Province, China
Center for Interventional Medical, The Fifth Affiliated Hospital, Sun Yat-sen University, Zhuhai, Guangdong Province, China

He Jian-Xing National Clinical Research Center for Respiratory Disease, Guangzhou Institute of Respiratory Health, First Affiliated Hospital of Guangzhou Medical University, State Key Laboratory of Respiratory Disease (Guangzhou Medical University), Guangzhou, China

http://orcid.org/0000-0002-0345-1945 Zheng Yong-Tang Key Laboratory of Animal Models and Human Disease Mechanisms of Chinese Academy of Sciences / Key Laboratory of Bioactive Peptides of Yunnan Province, Kunming Institute of Zoology, Chinese Academy of Sciences, Kunming, Yunnan 650223, China
Kunming National High-level Bio-safety Research Center for Non-human Primates, Center for Biosafety Mega-Science, Kunming Institute of Zoology, Chinese Academy of Sciences, Kunming, 650107, China

Wu Chung-I ChungIWu@scichina.org State Key Laboratory of Biocontrol, School of Life Sciences, Sun Yat-sen University, Guangzhou 510275, China

Corresponding author. E-mail: ChungIWu@scichina.orgThese authors contribute equally to this work.

Electronic publication date: 2020 Oct 12
Electronic Location ID: nwaa257
Copyright: © The Author(s) 2020. Published by Oxford University Press on behalf of China Science Publishing & Media Ltd.
Copyright year: 2020
License: This is an Open Access article distributed under the terms of the Creative Commons Attribution License (http://creativecommons.org/licenses/by/4.0/), which permits unrestricted reuse, distribution, and reproduction in any medium, provided the original work is properly cited.
License URL: https://creativecommons.org/licenses/by/4.0/

edited-state: accepted-manuscript
article-lifecycle: PAP

==============================
**Letter to Editor**

### A proposal for clinical trials of COVID-19 treatment using homo-harringtonine

Dear editor,

A scheme for treating COVID-19 was published in NSR in April (Wu and Wen 2020) based on blocking the translation of very large viral proteins. Since then, additional supporting data have greatly strengthened the proposal. The drug, HHT, is readily available and inexpensive as it has been approved since 2012 for treating leukemia. The *in vitro* and *in vivo* data clearly justify the planning of clinical trials. Given the specific mechanism, we believe a trial of modest scale would be sufficient to prove, or disprove, the efficacy of the treatment scheme. Such trials, however, are only feasible in regions with many new infections.

This letter, commenting on Wu and Wen (2020), has an expanded authorship that includes physicians with the first-hand experience in treating COVID-19 patients and virologists with recent publications on SARS-CoV-2. We wish to bring this proposal to the attention of the global community as there are few new cases in China for such trials.

### The original proposal

On April 15 2020, we published a proposal (Wu and Wen 2020) in NSR on a potential treatment of COVID-19. The proposal is based on the heightened activities of protein translation in two types of aberrant mammalian cells – cancer cells and virus-infected cells. The FDA approved drug, HHT (homo-harringtonine, or omacetaxine), has been known to disrupt the elongation step of protein translation with very high efficiency (Cephalotaxus Research Coordinating 1976; Fresno, et al. 1977; Garreau de Loubresse, et al. 2014). HHT, approved for treating leukemia since 2012 (Alvandi, et al. 2014), is probably the most powerful drug known to block protein translation.

There are several advantages to HHT over other drugs that block protein translation (Chen, et al. 2019; Liu, et al. 2018). The details can be found in Wu and Wen (2020) and a brief summary is provided below. 1) The making of a "super-protein" which will then yield 16 non-structural proteins by protease cleavage could be the Achilles heel of the coronaviruses including SARS-CoV-2. Since very few human proteins can rival this super-protein in size, it should be possible to preferentially disrupt the translation of viral proteins with minimal damages to the un-infected human cells. 2) Because HHT has been approved for clinical use since 2012, the safe dosage and toxicity effect are well understood. 3) The HHT dose required for suppressing virus proliferation is in the range of nano-molar concentration whereas the toxicity to human cells requires a concentration that is at least 30 times higher. 4) This low dose may permit several convenient means of drug delivery (see below).

Until very recently, glucocorticoids are the only intervention that reduces COVID-19-related death compared with the standard care. Three other drugs with modest efficacy are currently in use: hydroxychloroquine, remdesivir, and lopinavir-ritonavir (Siemieniuk, et al. 2020) in addition to the many published schemes (Cao, et al. 2020; Goldman, et al. 2020; Huang, et al. 2020; Hung, et al. 2020; Tang, et al. 2020; Wu and Poo 2020; Wu, et al. 2020; Zhang, et al. 2020). Given its well-known efficacy and safety, HHT deserves to enter clinical trials, along with other drugs announced to have the potential to block viral protein translation (Gordon, et al. 2020).

### The new evidence - in vitro and in vivo experiments

Although there was a sense of urgency for new treatment proposals, it was also apparent that the HHT scheme would need additional supports, which are provided here.

ORIGINAL UNEDITED MANUSCRIPT

© The Author(s) 2020. Published by Oxford University Press on behalf of China Science Publishing & Media Ltd. This is an Open Access article distributed under the terms of the Creative Commons Attribution License (http://creativecommons.org/licenses/by/4.0/), which permits unrestricted reuse, distribution, and reproduction in any medium, provided the original work is properly cited.

1) In vitro efficacy

The original proposal cited the efficacy of HHT on coronaviruses in general. We now provide new data on SARS-CoV-2. The $IC_{50}$ is between <100 – 350 nM depending on the conditions of the experiments and the lot of HHT used (Figure 1A-1C). It is also clear that, at 1 μM, the killing of the viruses is complete. As a comparison, remdesivir needs > 20 μM to achieve similar effect (Figure 1D). Hence, the efficacy of HHT in clearing SARS2-CoV-2 is in line with its efficacy against other coronaviruses (Andersen, et al. 2019; Cao, et al. 2015; Dong, et al. 2018; Kaur, et al. 2013). In a recent study, Choy et al. (2020) tested 16 compounds for SARS-CoV-2 inhibition and HHT stands out as well. (We notice that the concentration needed to achieve $EC_{50}$ is often much higher in Choy et al.'s data than in other experiments by 10 – 30 fold, although the relative efficacy among drugs is consistent with others.)

As the half-cytotoxic concentration ($CC_{50}$) of HHT in mammalian cells usually exceeds 10uM (Andersen, et al. 2019; Cao, et al. 2015; Kaur, et al. 2013), HHT has a selectivity index ($SI= CC_{50}/ IC_{50}$) > 30. Taken together, these results confirm the promises of HHT in treating COVID-19.

2) In vivo efficacy

In a preliminary experiment, we infected ACE2 humanized mice with SARS-CoV-2. This inbred mice model was generated by integrating CRISPR-Cas9 and tetraploid complementation. The infected mice were either untreated as the control or treated by intraperitoneal injection of 40μg HHT in 100μl normal saline. The first dose is administered 2 hours before the virus challenge. Since HHT inhibits viral replication but does not block virus entry, the lead time of 2 hours should be sufficient for drug diffusion into the cells. HHT is administered daily for 3 days.

On the third day post infection (d.p.i), mice were sacrificed and SARS-CoV-2 genomic RNA in lung tissues was measured by qRT-PCR. Among the 3 untreated mice, we obtained from 15 samples (five lobes of the lung from each mouse) the viral load of each sample, classified as ND (non-detectable), background (<10^2 copies/μg total RNA) or high load (10^5 – 10^8). We should note that the infected mice, if untreated, would start to clear the viral load starting on the fourth day. The distribution of viral load, summarized as [ND, Low, High], was [3, 6, 6] for the control. In contrast, the distribution for the 3 HHT-treated mice was [9, 6, 0]. Although the sample size was small, HHT did repress the viral load to the background level in all 15 samples whereas the load was high in 6 of the 15 untreated samples. Given all the evidence, we believe that clinical trials should be the next step as suggested below.

**Proposed clinical trial – I. The pre-treatment**

From the standpoint of public health, the goal is to stop the spread of the virus and the progression of the infection, *before* the infection is confirmed. Here, we propose a two-step scheme aiming at the clearance of the viral load in the early phases of COVID infection. Late phases of infection with severe organ damages are not the target stages. For individuals who are not yet confirmed for infection but have reasons to suspect the possibility (such as close contacts with positive cases), we now recommend a pre-treatment procedure as follows.

First, as shown by Wolfer et al. (Wölfel, et al. 2020) and He et al. (He, et al. 2020), SARS-CoV-2 viruses may spend the first four days, on average, in the throat and nasal passage before they enter the lower respiratory tract. Second, the WHO guideline (Kratzel, et al. 2020) has shown that SARS-CoV-2 virus cannot survive for more than 30 seconds in 30% alcohol. Since many hard liquor and spirits have an alcohol content of above 30%, gargling 2-3 times a day with such hard liquor, each time for 30 seconds, might be effective in substantially arresting disease progression as well as inhibiting the spread of the viruses to others.

This pre-treatment procedure is not likely to impair health (even if gargling is followed by swallowing, which should be discouraged). Furthermore, there have been various non-scientific indications of alcohol's efficacy in reducing the viral load during COVID-19 (Sattar, et al. 2020). Therefore, a large-scale clinical

ORIGINAL UNEDITED MANUSCRIPT

trial should be possible with little social and medical costs. If the disease symptoms do progress, then the HHT treatment would be an option.

**Proposed clinical trial – II. The HHT delivery**

If the pre-treatment fails to clear the virus, an option would be the standard delivery of HHT by intravenous injection, which lasts at least 3 hours each day in a course of 4-6 days in treating Leukemia over several courses.

1) Intravenous injection – The safety level of HHT by intravenous injection is well known.

i) In vitro dosage – Human HEK-293T cells can survive the 20 μM treatment for > 3 hours or 5 μM for > 24 hours. Indeed, 5 μM is far higher than necessary for suppressing SARS-CoV-2.

ii) Dosage for the HHT treatment of leukemia - 1.5 mg/m^2 (about 2.5 mg/[60kg person] or 100nM for adult patients) with daily continuous intravenous infusion for 3-6 hours up to 28 days.

iii) It has been reported that the maximum tolerated and therapeutically effective dose of HHT for treating adult leukemia patients is 4mg daily for 14 days (Kantarjian, et al. 2001). The treatment duration for COVID-19 is likely to be much shorter.

2) Nebulization – An alternative delivery would be by nebulization although the safety on humans has to be evaluated. There are reasons for preferring nebulization to intravenous injection in reducing the viral load and stopping the progression of symptoms. With nebulization, the drug concentration should be higher in the lungs than in other tissues, but probably for no more than a few hours after nebulization (Lemarie, et al. 2011). The drug would then be distributed through blood circulation as in IV.

In previous reports, HHT delivered by subcutaneous injection can only achieve a systemic concentration of 50 – 60 nM while intravenous injection may achieve a higher concentration of 100-200 nM (Chan, et al. 1989; Nemunaitis, et al. 2013). The HHT concentration achievable by intravenous injection is very close to the in vitro $IC_{50}$ at 100-300 nM, thus leaving almost no margin for errors. Furthermore, nebulization can be more easily administered by the patients themselves than IV when multiple applications per day are called for. A caveat is that aerosols generated during nebulization should be strictly regulated to avoid viral spread.

Since the toxicity by nebulization, especially to the lungs, is not known, we have assessed the effect of HHT nebulization on two dogs, each weighing ~6kg. Dogs may be the only animal model on which nebulization can be applied. The dosage was ramped up from 0. 2 mg to 0.4, 0.6, 0.8, and 1mg per day. Each dosage was applied in two successive days with the whole treatment lasting 10 days. Each dog is monitored by daily physical assessment, complete blood count, chemistry profile, as well as a chest X-ray examination every two days. No toxicological changes related to the treatment was observed. Since the nebulization treatment in humans can be delivered evenly in the course of a day, 1 mg of HHT for humans should be well tolerated for several days.

Recommended parameters:

If the goal is to sustain the higher concentration of HHT in the lungs, we would recommend several episodes of nebulization per day (see also the next section). Based on the dosage in treating leukemia of a person of 60 kg, we suggest 1 mg per day in 15 ml saline by nebulization 5 times a day at the 4-hour interval, allowing 8 hours sleep. Each episode would be 15 minutes to nebulize 3 ml of solution. The total dose may be gradually ramped up to 4 mg per day if safety permits.

The recommendation is based on our knowledge of sustaining the HHT concentration in the lungs. The optimal treatment cycles can only be found empirically. Nevertheless, given the in vitro and in vivo data, the scale of the clinical trial can be delineated. If the scheme works as expected, the nebulization of HHT should show a strong reduction in the viral load of several orders of magnitude within a few days. We estimate that

ORIGINAL UNEDITED MANUSCRIPT

a clinical trial of a modest number of patients (10 - 50) should be sufficient to prove or dis-prove the efficacy of HHT.

**Final remarks**

The main obstacle for the HHT clinical trials in many countries is the very low numbers of new COVID-19 patients. At the same time, medical facilities elsewhere may have an acute need for treating new COVID-19 patients by reducing their viral loads. For these facilities, the proposed scheme here may be an option for small-scale clinical trials.

**Acknowledgements**

We are grateful to Chen Hualan, Zhong Gongxun, Jiang Chengyu, Shi Suhua, Zhao Yong, He Xionglei, Cao Yongchang, and Zhang Yaping for helpful comments. This work was supported by the National Natural Science Foundation of China (91731000, 31730046 and 81972691), Guangdong Basic and Applied Basic Research Foundation (2020B1515020030), National Key Research and Development Project (2020YFC0847000, 2020YFC0842000).

Hai-Jun Wen[1 †], Feng-Liang Liu[2,3 †], Ming-Xing Huang[4 †], Rong-Hua Luo[2,3], Wen-Bin He[5,6], Jing Feng[5,6], Fang-Liang Chen[7], Qi-Chun Cai[8], Hua-Juan Ma[8], Zi-Feng Yang[9,10], Xi Zhou[11,12,13], You Shang[13,14], Xue-Mei Lyu[5,6], Ding-Yu Zhang[12,13], Fei Xiao[4,15], Hong Shan[15,16], Jian-Xing He[9], Yong-Tang Zheng[2,3], and Chung-I Wu[1] *

1. State Key Laboratory of Biocontrol, School of Life Sciences, Sun Yat-sen University, Guangzhou 510275, China
2. Key Laboratory of Animal Models and Human Disease Mechanisms of Chinese Academy of Sciences / Key Laboratory of Bioactive Peptides of Yunnan Province, Kunming Institute of Zoology, Chinese Academy of Sciences, Kunming, Yunnan 650223, China
3. Kunming National High-level Bio-safety Research Center for Non-human Primates, Center for Biosafety Mega-Science, Kunming Institute of Zoology, Chinese Academy of Sciences, Kunming, 650107, China
4. Department of Infectious Diseases, The Fifth Affiliated Hospital, Sun Yat-sen University, Zhuhai, Guangdong, China
5. State Key Laboratory of Genetic Resources and Evolution, Kunming Institute of Zoology, Chinese Academy of Science, Kunming 650223, China
6. Center for Excellence in Animal Evolution and Genetics, Chinese Academy of Sciences, Kunming 650223, China
7. Kunming Police Dog Base of the Ministry of Public Security, Kunming, China
8. Cancer Center, Clifford Hospital, Jinan University, Guangzhou, PR China
9. National Clinical Research Center for Respiratory Disease, Guangzhou Institute of Respiratory Health, First Affiliated Hospital of Guangzhou Medical University, State Key Laboratory of Respiratory Disease (Guangzhou Medical University), Guangzhou, China
10. Faculty of Chinese Medicine, Macau University of Science and Technology, Taipa, Macau SAR, PR China
11. State Key Laboratory of Virology, Wuhan Institute of Virology, Center for Biosafety Mega-Science, Chinese Academy of Sciences (CAS), China
12. Center for Translational Medicine, Wuhan Jinyintan Hospital, China
13. Joint Laboratory of Infectious Diseases and Health, Wuhan Institute of Virology & Wuhan Jinyintan Hospital, CAS, China
14. Department of Critical Care Medicine, Union Hospital, Tongji Medical College, Huazhong University of Science and Technology, Wuhan, China
15. Guangdong Provincial Key Laboratory of Biomedical Imaging and Guangdong Provincial Engineering Research Center of Molecular Imaging, The Fifth Affiliated Hospital, Sun Yat-sen University, Zhuhai, Guangdong Province, China

ORIGINAL UNEDITED MANUSCRIPT

16. Center for Interventional Medical, The Fifth Affiliated Hospital, Sun Yat-sen University, Zhuhai, Guangdong Province, China

†These authors contribute equally to this work.
*Correspondence to:
Chung-I Wu, ChungIWu@scichina.org

**References**

Alvandi F, Kwitkowski VE, Ko C-W, Rothmann MD, Ricci S, Saber H, Ghosh D, Brown J, Pfeiler E, Chikhale E, Grillo J, Bullock J, Kane R, Kaminskas E, Farrell AT, Pazdur R 2014. U.S. Food and Drug Administration approval summary: omacetaxine mepesuccinate as treatment for chronic myeloid leukemia. The oncologist 19: 94-99. doi: 10.1634/theoncologist.2013-0077

Andersen PI, Krpina K, Ianevski A, Shtaida N, Jo E, Yang J, Koit S, Tenson T, Hukkanen V, Anthonsen MW, Bjoras M, Evander M, Windisch MP, Zusinaite E, Kainov DE 2019. Novel Antiviral Activities of Obatoclax, Emetine, Niclosamide, Brequinar, and Homoharringtonine. Viruses 11. doi: 10.3390/v11100964

Cao B, Wang Y, Wen D, Liu W, Wang J, Fan G, Ruan L, Song B, Cai Y, Wei M, Li X, Xia J, Chen N, Xiang J, Yu T, Bai T, Xie X, Zhang L, Li C, Yuan Y, Chen H, Li H, Huang H, Tu S, Gong F, Liu Y, Wei Y, Dong C, Zhou F, Gu X, Xu J, Liu Z, Zhang Y, Li H, Shang L, Wang K, Li K, Zhou X, Dong X, Qu Z, Lu S, Hu X, Ruan S, Luo S, Wu J, Peng L, Cheng F, Pan L, Zou J, Jia C, Wang J, Liu X, Wang S, Wu X, Ge Q, He J, Zhan H, Qiu F, Guo L, Huang C, Jaki T, Hayden FG, Horby PW, Zhang D, Wang C 2020. A Trial of Lopinavir–Ritonavir in Adults Hospitalized with Severe Covid-19. New England Journal of Medicine 382: 1787-1799. doi: 10.1056/NEJMoa2001282

Cao J, Forrest JC, Zhang X 2015. A screen of the NIH Clinical Collection small molecule library identifies potential anti-coronavirus drugs. Antiviral Res 114: 1-10. doi: 10.1016/j.antiviral.2014.11.010

Cephalotaxus Research Coordinating G 1976. Cephalotaxine esters in the treatment of acute leukemia. A preliminary clinical assessment. Chin Med J 2: 263-272.

Chan Y-PM, Lee F-W, Siu T-SS 1989. Quantitation of homoharringtonine in plasma by high-performance liquid chromatography with amperometric detection. Journal of Chromatography B: Biomedical Sciences and Applications 496: 155-166. doi: https://doi.org/10.1016/S0378-4347(00)82562-1

Chen Y, Shen Y, Lin P, Tong D, Zhao Y, Allesina S, Shen X, Wu C-I 2019. Gene regulatory network stabilized by pervasive weak repressions: microRNA functions revealed by the May–Wigner theory. National Science Review 6: 1176-1188. doi: 10.1093/nsr/nwz076

Dong HJ, Wang ZH, Meng W, Li CC, Hu YX, Zhou L, Wang XJ 2018. The Natural Compound Homoharringtonine Presents Broad Antiviral Activity In Vitro and In Vivo. Viruses 10. doi: 10.3390/v10110601

Fresno M, Jiménez A, Vázquez D 1977. Inhibition of translation in eukaryotic systems by harringtonine. European journal of biochemistry 72: 323-330. doi: 10.1111/j.1432-1033.1977.tb11256.x

Garreau de Loubresse N, Prokhorova I, Holtkamp W, Rodnina MV, Yusupova G, Yusupov M 2014. Structural basis for the inhibition of the eukaryotic ribosome. Nature 513: 517-522. doi: 10.1038/nature13737

Goldman JD, Lye DCB, Hui DS, Marks KM, Bruno R, Montejano R, Spinner CD, Galli M, Ahn M-Y, Nahass RG, Chen Y-S, SenGupta D, Hyland RH, Osinusi AO, Cao H, Blair C, Wei X, Gaggar A, Brainard DM, Towner WJ, Muñoz J, Mullane KM, Marty FM, Tashima KT, Diaz G, Subramanian A 2020. Remdesivir for 5 or 10 Days in Patients with Severe Covid-19. New England Journal of Medicine. doi: 10.1056/NEJMoa2015301

Gordon DE, Jang GM, Bouhaddou M, Xu J, Obernier K, White KM, O'Meara MJ, Rezelj VV, Guo JZ, Swaney DL, Tummino TA, Huettenhain R, Kaake RM, Richards AL, Tutuncuoglu B, Foussard H, Batra J, Haas K, Modak M, Kim M, Haas P, Polacco BJ, Braberg H, Fabius JM, Eckhardt M, Soucheray M, Bennett MJ, Cakir M, McGregor MJ, Li Q, Meyer B, Roesch F, Vallet T, Mac Kain A, Miorin L, Moreno E, Naing ZZC, Zhou Y, Peng S, Shi Y, Zhang Z, Shen W, Kirby IT, Melnyk JE, Chorba JS, Lou K, Dai SA, Barrio-

ORIGINAL UNEDITED MANUSCRIPT

Hernandez I, Memon D, Hernandez-Armenta C, Lyu J, Mathy CJP, Perica T, Pilla KB, Ganesan SJ, Saltzberg DJ, Rakesh R, Liu X, Rosenthal SB, Calviello L, Venkataramanan S, Liboy-Lugo J, Lin Y, Huang XP, Liu Y, Wankowicz SA, Bohn M, Safari M, Ugur FS, Koh C, Savar NS, Tran QD, Shengjuler D, Fletcher SJ, O'Neal MC, Cai Y, Chang JCJ, Broadhurst DJ, Klippsten S, Sharp PP, Wenzell NA, Kuzuoglu D, Wang HY, Trenker R, Young JM, Cavero DA, Hiatt J, Roth TL, Rathore U, Subramanian A, Noack J, Hubert M, Stroud RM, Frankel AD, Rosenberg OS, Verba KA, Agard DA, Ott M, Emerman M, Jura N, von Zastrow M, Verdin E, Ashworth A, Schwartz O, d'Enfert C, Mukherjee S, Jacobson M, Malik HS, Fujimori DG, Ideker T, Craik CS, Floor SN, Fraser JS, Gross JD, Sali A, Roth BL, Ruggero D, Taunton J, Kortemme T, Beltrao P, Vignuzzi M, Garcia-Sastre A, Shokat KM, Shoichet BK, Krogan NJ 2020. A SARS-CoV-2 protein interaction map reveals targets for drug repurposing. Nature. doi: 10.1038/s41586-020-2286-9

He X, Lau EHY, Wu P, Deng X, Wang J, Hao X, Lau YC, Wong JY, Guan Y, Tan X, Mo X, Chen Y, Liao B, Chen W, Hu F, Zhang Q, Zhong M, Wu Y, Zhao L, Zhang F, Cowling BJ, Li F, Leung GM 2020. Temporal dynamics in viral shedding and transmissibility of COVID-19. Nature Medicine. doi: 10.1038/s41591-020-0869-5

Huang M, Li M, Xiao F, Liang J, Pang P, Tang T, Liu S, Chen B, Shu J, You Y, Li Y, Tang M, Zhou J, Jiang G, Xiang J, Hong W, He S, Wang Z, Feng J, Lin C, Ye Y, Wu Z, Li Y, Zhong B, Sun R, Hong Z, Liu J, Chen H, Wang X, Li Z, Pei D, Tian L, Xia J, Jiang S, Zhong N, Shan H 2020. Preliminary evidence from a multicenter prospective observational study of the safety and efficacy of chloroquine for the treatment of COVID-19. medRxiv: 2020.2004.2026.20081059. doi: 10.1101/2020.04.26.20081059

Hung IF-N, Lung K-C, Tso EY-K, Liu R, Chung TW-H, Chu M-Y, Ng Y-Y, Lo J, Chan J, Tam AR, Shum H-P, Chan V, Wu AK-L, Sin K-M, Leung W-S, Law W-L, Lung DC, Sin S, Yeung P, Yip CC-Y, Zhang RR, Fung AY-F, Yan EY-W, Leung K-H, Ip JD, Chu AW-H, Chan W-M, Ng AC-K, Lee R, Fung K, Yeung A, Wu T-C, Chan JW-M, Yan W-W, Chan W-M, Chan JF-W, Lie AK-W, Tsang OT-Y, Cheng VC-C, Que T-L, Lau C-S, Chan K-H, To KK-W, Yuen K-Y 2020. Triple combination of interferon beta-1b, lopinavir-ritonavir, and ribavirin in the treatment of patients admitted to hospital with COVID-19: an open-label, randomised, phase 2 trial. The Lancet. doi: 10.1016/S0140-6736(20)31042-4

Kantarjian HM, Talpaz M, Santini V, Murgo A, Cheson B, O'Brien SM 2001. Homoharringtonine: history, current research, and future direction. Cancer 92: 1591-1605. doi: 10.1002/1097-0142(20010915)92:6<1591::aid-cncr1485>3.0.co;2-u

Kaur P, Thiruchelvan M, Lee RC, Chen H, Chen KC, Ng ML, Chu JJ 2013. Inhibition of chikungunya virus replication by harringtonine, a novel antiviral that suppresses viral protein expression. Antimicrob Agents Chemother 57: 155-167. doi: 10.1128/AAC.01467-12

Kratzel A, Todt D, V'kovski P, Steiner S, Gultom ML, Thao TTN, Ebert N, Holwerda M, Steinmann J, Niemeyer D, Dijkman R, Kampf G, Drosten C, Steinmann E, Thiel V, Pfaender S 2020. Efficient inactivation of SARS-CoV-2 by WHO-recommended hand rub formulations and alcohols. bioRxiv: 2020.2003.2010.986711. doi: 10.1101/2020.03.10.986711

Lemarie E, Vecellio L, Hureaux J, Prunier C, Valat C, Grimbert D, Boidron-Celle M, Giraudeau B, le Pape A, Pichon E, Diot P, el Houfia A, Gagnadoux F 2011. Aerosolized Gemcitabine in Patients with Carcinoma of the Lung: Feasibility and Safety Study. Journal of Aerosol Medicine and Pulmonary Drug Delivery 24: 261-270. doi: 10.1089/jamp.2010.0872

Liu X, Chang X, Leng S, Tang H, Aihara K, Chen L 2018. Detection for disease tipping points by landscape dynamic network biomarkers. National Science Review 6: 775-785. doi: 10.1093/nsr/nwy162

Nemunaitis J, Mita A, Stephenson J, Mita MM, Sarantopoulos J, Padmanabhan-Iyer S, Nanda N, Gleich L, Benichou A-C, Craig A 2013. Pharmacokinetic study of omacetaxine mepesuccinate administered subcutaneously to patients with advanced solid and hematologic tumors. Cancer Chemotherapy and Pharmacology 71: 35-41. doi: 10.1007/s00280-012-1963-2

Sattar SA, Bosch A, Wise MP, Fegan C, Wakelam MJO, Humphreys I, Jones SA, Murphy RC, Maillard J-Y, Stanton R, Thomas D, O'Donnell VB 2020. Potential Role of Oral Rinses Targeting the Viral Lipid Envelope in SARS-CoV-2 Infection. Function 1. doi: 10.1093/function/zqaa002

Siemieniuk RA, Bartoszko JJ, Ge L, Zeraatkar D, Izcovich A, Pardo-Hernandez H, Rochwerg B, Lamontagne F, Han MA, Kum E, Liu Q, Agarwal A, Agoritsas T, Alexander P, Chu DK, Couban R, Darzi A, Devji T, Fang B, Fang C, Flottorp SA, Foroutan F, Heels-Ansdell D, Honarmand K, Hou L, Hou X, Ibrahim Q, Loeb M, Marcucci M, McLeod SL, Motaghi S, Murthy S, Mustafa RA, Neary JD, Qasim A, Rada G, Riaz

ORIGINAL UNEDITED MANUSCRIPT

IB, Sadeghirad B, Sekercioglu N, Sheng L, Switzer C, Tendal B, Thabane L, Tomlinson G, Turner T, Vandvik PO, Vernooij RW, Viteri-Garcia A, Wang Y, Yao L, Ye Z, Guyatt GH, Brignardello-Petersen R 2020. Drug treatments for covid-19: living systematic review and network meta-analysis. BMJ 370: m2980. doi: 10.1136/bmj.m2980
Tang W, Cao Z, Han M, Wang Z, Chen J, Sun W, Wu Y, Xiao W, Liu S, Chen E, Chen W, Wang X, Yang J, Lin J, Zhao Q, Yan Y, Xie Z, Li D, Yang Y, Liu L, Qu J, Ning G, Shi G, Xie Q 2020. Hydroxychloroquine in patients with mainly mild to moderate coronavirus disease 2019: open label, randomised controlled trial. BMJ 369: m1849. doi: 10.1136/bmj.m1849
Wölfel R, Corman VM, Guggemos W, Seilmaier M, Zange S, Müller MA, Niemeyer D, Jones TC, Vollmar P, Rothe C, Hoelscher M, Bleicker T, Brünink S, Schneider J, Ehmann R, Zwirglmaier K, Drosten C, Wendtner C 2020. Virological assessment of hospitalized patients with COVID-2019. Nature. doi: 10.1038/s41586-020-2196-x
Wu C-I, Poo M-m 2020. Moral imperative for immediate release of 2019-nCoV sequence data. National Science Review 7: 719-720. doi: 10.1093/nsr/nwaa030
Wu C-I, Wen H 2020. Heightened protein-translation activities in mammalian cells and the disease/treatment implications. National Science Review. doi: 10.1093/nsr/nwaa066
Wu D, Shu T, Yang X, Song J-X, Zhang M, Yao C, Liu W, Huang M, Yu Y, Yang Q, Zhu T, Xu J, Mu J, Wang Y, Wang H, Tang T, Ren Y, Wu Y, Lin S-H, Qiu Y, Zhang D-Y, Shang Y, Zhou X 2020. Plasma Metabolomic and Lipidomic Alterations Associated with COVID-19. National Science Review. doi: 10.1093/nsr/nwaa086
Zhang B, Liu S, Tan T, Huang W, Dong Y, Chen L, Chen Q, Zhang L, Zhong Q, Zhang X, Zou Y, Zhang S 2020. Treatment With Convalescent Plasma for Critically Ill Patients With Severe Acute Respiratory Syndrome Coronavirus 2 Infection. Chest. doi: https://doi.org/10.1016/j.chest.2020.03.039

ORIGINAL UNEDITED MANUSCRIPT

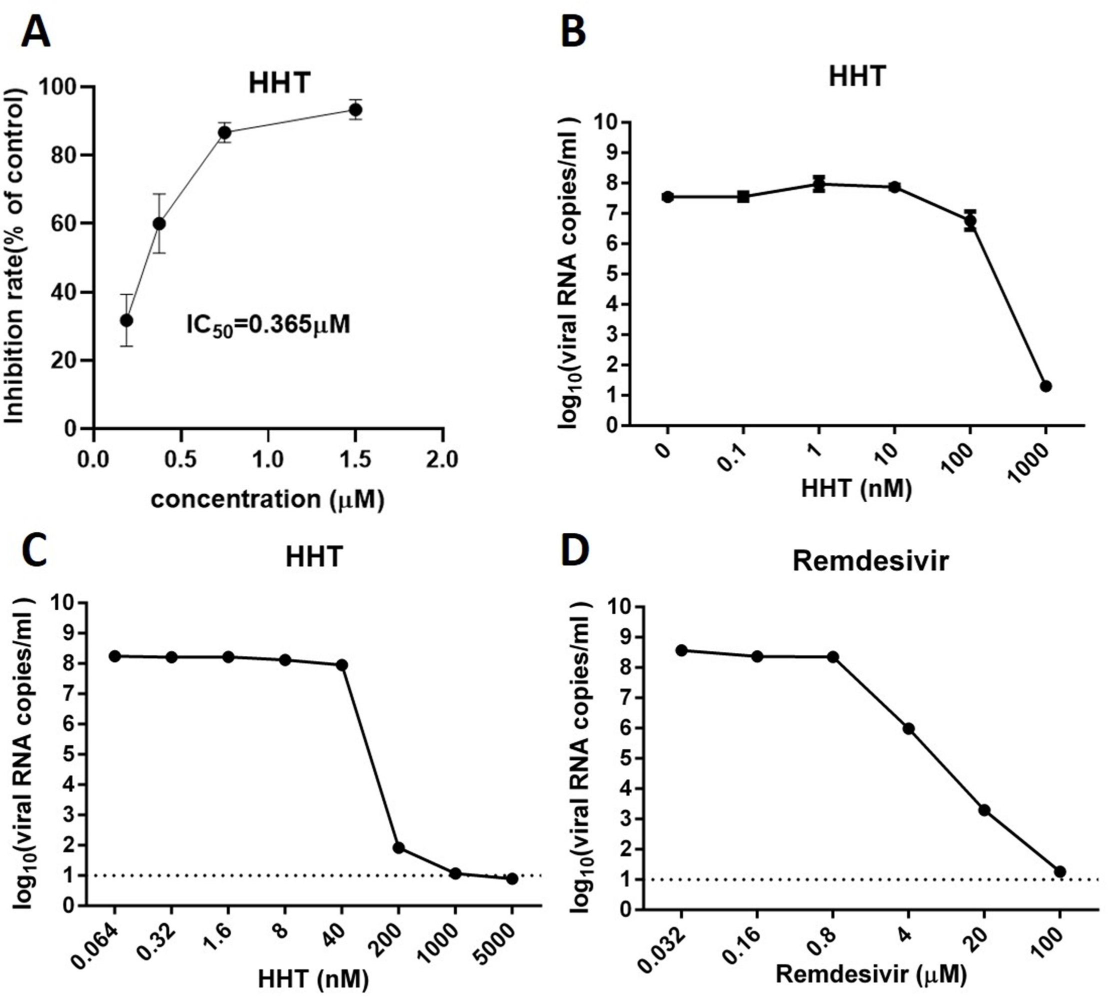

Figure 1. HHT inhibition of SARS-CoV-2 replication in vitro as performed by various laboratories, including (A) State Key Laboratory of Respiratory Disease (Guangzhou Medical University); (B) Harbin Veterinary Research Institute; and (C) Kunming Institute of Zoology. (D)For a comparison, the inhibitory effect of Remdesivir on SARS-CoV-2 was also assayed in Kunming Institute of Zoology. The horizontal dash line in (C) and (D) indicated complete inhibition against SARS-CoV-2. At 1 μM, HHT can clear the virus whereas Remdesivir would require 20 – 100 μM to have the same effect.

ORIGINAL UNEDITED
RIPT
